# Evidence for Ecological Flexibility in the Cosmopolitan Genus *Curtobacterium*

**DOI:** 10.3389/fmicb.2016.01874

**Published:** 2016-11-22

**Authors:** Alexander B. Chase, Philip Arevalo, Martin F. Polz, Renaud Berlemont, Jennifer B. H. Martiny

**Affiliations:** ^1^Department of Ecology and Evolutionary Biology, University of California, IrvineIrvine, CA, USA; ^2^Parsons Laboratory for Environmental Science and Engineering, Massachusetts Institute of TechnologyCambridge, MA, USA; ^3^Department of Biological Sciences, California State University Long BeachLong Beach, CA, USA

**Keywords:** decomposition, leaf litter, glycoside hydrolases, Microbacteriaceae, Actinobacteria

## Abstract

Assigning ecological roles to bacterial taxa remains imperative to understanding how microbial communities will respond to changing environmental conditions. Here we analyze the genus *Curtobacterium*, as it was found to be the most abundant taxon in a leaf litter community in southern California. Traditional characterization of this taxon predominantly associates it as the causal pathogen in the agricultural crops of dry beans. Therefore, we sought to investigate whether the abundance of this genus was because of its role as a plant pathogen or another ecological role. By collating >24,000 16S rRNA sequences with 120 genomes across the Microbacteriaceae family, we show that *Curtobacterium* has a global distribution with a predominant presence in soil ecosystems. Moreover, this genus harbors a high diversity of genomic potential for the degradation of carbohydrates, specifically with regards to structural polysaccharides. We conclude that *Curtobacterium* may be responsible for the degradation of organic matter within litter communities.

## Introduction

Traditional ecological characterization of microorganisms often narrowly defines their roles in terms of interspecies interactions. Such limited classification of interactions ignores the dynamic alterations of life cycles indicative of microorganisms in changing environmental conditions (Redman et al., 2001; Kogel et al., 2006; Newton et al., 2010). Depending on the environment, microbes can transition from symbiont to pathogen (Johnson et al., 1997) or drastically alter their life history strategy altogether. For instance, endophytic fungi transition to decomposers after the leaves fall off its host plant (Osono, 2006; Korkama-Rajala et al., 2008). Such flexibility in ecological roles may also explain why *Curtobacterium*, a bacterial genus traditionally viewed as a plant pathogen (Hsieh et al., 2005), was recently found to be the dominant bacterium in the leaf litter of a Mediterranean-like grassland community (Matulich et al., 2015).

Members of the *Curtobacterium* genus are Gram-positive, obligately aerobic chemoorganotrophs in the family Microbacteriaceae, phylum Actinobacteria (Evtushenko and Takeuchi, 2006). The habitat of *Curtobacterium* is described mainly in association with plants and especially, the phyllosphere (Komagata et al., 1965; Behrendt et al., 2002). Indeed, most studies investigating *Curtobacterium* focus on its role as an economically important plant pathogen (Huang et al., 2009; Osdaghi et al., 2015b). The best-studied pathovar, *C. flaccumfaciens* pv. *flaccumfaciens*, is the causal agent of bacterial wilt in dry beans worldwide with reports on five continents (Wood and Easdown, 1990; Harveson et al., 2006; EPPO, 2011; Soares et al., 2013; Osdaghi et al., 2015a). The disease harbors a high degree of genetic and phenotypic diversity (Hedges, 1926; Conner et al., 2008) even within a single host (Agarkova et al., 2012).

Although economically important, *C. flaccumfaciens* is the only species of *Curtobacterium* associated with plant pathogenesis (Young et al., 1996), and there is evidence that other *Curtobacterium* species perform other ecological roles. For instance, isolates have been identified as endophytic symbionts (Sturz et al., 1997, 1999; Elbeltagy et al., 2000; Araújo et al., 2001; Bulgari et al., 2009). Similar to other beneficial endophytes (Benhamou et al., 2000; Taghavi et al., 2009), *Curtobacterium* can elicit plant defense responses (Bulgari et al., 2011) and reduce disease symptoms (Lacava et al., 2007). The genus has also been found to associate with roots and promote plant growth (Sturz et al., 1997). Even the presence of *C. flaccumfaciens* in the rhizosphere induced a systematic resistance in cucumber plants to pathogens (Raupach and Kloepper, 1998) and promoted plant growth (Raupach and Kloepper, 2000). *Curtobacterium* can also be found in soil (Ohya et al., 1986; Aizawa et al., 2007; Kim et al., 2008) with an ability to persist on plant debris (Silva Junior et al., 2012), although as a non-spore forming bacterium, the genus might be assumed to be a poor survivor in soil (Vidaver, 1982).

Our previous work in a Mediterranean-like grassland community revealed that a *Curtobacterium* taxon (defined by ≥ 97% similarity in 16S rRNA sequence) was the most abundant bacterium in leaf litter, the top layer of soil. The leaf litter community at this site is dominated by bacteria with a bacteria to fungi biomass ratio up to 30:1 (Alster et al., 2013). The community is highly diverse, but uneven; three phyla (Actinobacteria, Bacteroidetes, and Proteobacteria) made up 95% of total bacterial abundance (Matulich et al., 2015). Further analysis revealed that *Curtobacterium* constituted ~18% of 16S rRNA sequences amplified directly from 177 litter samples over a two-year period (Matulich et al., 2015). This high abundance was further supported by sequenced metagenomes from the same grassland. These samples suggested that >8% of the reads fall within Microbacteriaceae (Berlemont et al., 2014), most likely an underestimate due to lack of representation of *Curtobacterium* in genomic databases.

Given its dominance in grassland litter, this current study investigates the potential for *Curtobacterium* to play ecological roles other than a plant pathogen, and in particular, as a decomposer. We asked: (1) What is the geographic and habitat distribution of the genus? (2) Is the phylogenetic diversity of *Curtobacterium* related to its habitat distribution? and (3) What is the genus' genomic potential to degrade recalcitrant carbohydrates? To address these questions, we isolated and sequenced 14 *Curtobacterium* strains from grassland litter. Then, we combined our genome sequences with publically-available sequences from a variety of habitats and locations, collating >24,000 Microbacteriaceae 16S rRNA sequences. Finally, we investigated the genomic diversity of *Curtobacterium* with regards to its ability to degrade carbohydrates, an important attribute for litter decomposition. We searched for glycoside hydrolases (GHs), enzymes that target specific glycosidic bonds of carbohydrates (including cellulose and xylan in plant cell walls). We conclude that the genus *Curtobacterium* is cosmopolitan in terrestrial ecosystems and may be, at an intrageneric level, involved in a variety of ecological roles including decomposition of organic matter.

## Materials and methods

### Geographic distribution

To investigate the geographic extent of *Curtobacterium*, we searched for *Curtobacterium* sequences within the open reference dataset of the Earth Microbiome Project (EMP) (Gilbert et al., 2014). We obtained 41 unique *Curtobacterium* OTUs with metadata from 14,096 uploaded samples.

To gather additional *Curtobacterium* sequences, we used BLAST to search for sequences similar to eight *Curtobacterium* 16S rRNA gene sequences from the GreenGenes “Core Set” database (DeSantis et al., 2006) against the GenBank nr database (Benson et al., 2008). Additional sequences were identified using the keyword search: “Microbacteriaceae Curtobacterium 16S ribosomal RNA gene.” After removing redundant entries and 16S rRNA sequences that could not be identified to the genus level, 11,484 unique sequences remained.

We extracted metadata from either corresponding GenBank files, the EMP 10k merged mapping file, or manually reviewed the published literature to identify the isolation source and location of all retrieved *Curtobacterium* sequences. Each sequence was assigned to one of seven ecosystems: animal microbiome, aquatic, artificial, atmosphere, human microbiome, ice, or terrestrial. Terrestrial samples were further divided into six categories: plant, plant roots, plant seeds, rock, sediment, and soil.

The geographic distribution of the EMP and GenBank sequences were plotted using the R library “rworldmap” (South, 2011). For samples with minimal location data (mainly from the GenBank dataset), we used a publicly available dataset from Google Developers^1^ to assign approximate longitude and latitude coordinates based on the state, providence, and/or country of origin.

### Phylogenetic diversity

To establish a robust phylogenetic distribution, we downloaded 16S rRNA gene sequences from the SILVA SSU r123 database (Pruesse et al., 2007) on August 6, 2015. Sequences were obtained using SILVA's assigned taxonomy, yielding 1519 *Curtobacterium* sequences and 24,835 Microbacteriaceae sequences. Due to variability in taxonomic nomenclature by various databases, we confirmed all taxonomic assignments of all downloaded sequences. First, we assigned taxonomy with QIIME v1.6 (Caporaso et al., 2010) using the UCLUST consensus taxonomy assigner (Edgar, 2010) against the GreenGenes reference database (May 2013 revision; DeSantis et al., 2006). Next, we compared these taxonomic assignments to those assigned using the RDP Classifier (Wang et al., 2007). After removing sequences incorrectly assigned to *Curtobacterium* and/or Microbacteriaceae and other low quality sequences (<80% identity, <700 bp), 12,469 sequences remained.

To select a subset of this sequence diversity for phylogenetic analysis, we clustered the filtered sequences and the sequences of our litter isolates (see below) using QIIME v1.9. We defined OTUs at 97% identity with UCLUST using the optimal flag for OTU picking, and selected representative sequences for each OTU. The representative sequences were assigned a taxonomic designation at the genus level using a combination of UCLUST, BLAST, and the RDP Classifier. Specifically, genera designations for the representative sequences were only assigned when at least two of the aforementioned taxonomic designations matched at the genus level. We aligned the sequences using the Infernal Alignment Tool (Nawrocki et al., 2009). Gaps common in >90% of aligned sequences were manually removed, resulting in a 1900 bp alignment. OTU representative sequences that contained >25% gap regions were also removed. As a result, the sequences obtained from the EMP database were too short (~100–250 bp; mean size = 134 bp) to integrate in the phylogeny with the full 16S rRNA gene obtained from other datasets. A maximum likelihood tree with 100 bootstrap replications was constructed with RAxML v8.0, using the GTR + Gamma distribution model (Stamatakis, 2014). The tree was visualized using the Interactive Tree of Life (iTOL; Letunic and Bork, 2007).

The pipeline above was modified slightly to investigate the phylogenetic diversity within the *Curtobacterium* genus. This analysis incorporated all available 16S rRNA genes (*n* = 1532) from GenBank, SILVA, and litter isolates assigned to *Curtobacterium*. OTUs were clustered at 99% similarity to provide finer taxonomic resolution and included a sister genus, *Frigoribacterium*, as an outgroup.

### Genomic analysis of litter isolates

#### Isolation and identification of litter isolates

Bacteria from litter were isolated from two grassland global change experiments. Isolates from the Loma Ridge Global Change Experiment (LRGCE) (in Irvine, California, USA [33° 44′ N, 117° 42′ W]; Potts et al., 2012) were previously identified and presented in Mouginot et al. (2014). Briefly, leaf litter particles were suspended in saline and inoculated onto nutrient-limited media plates made from Loma Ridge litter leachate and incubated at room temperature. For this study, additional strains were isolated from the Boston-Area Climate Experiment (BACE) [42° 23′ N, 71° 12′ W] (Tharayil et al., 2011) using the same protocol on Boston litter leachate media. Individual colonies were streaked onto LB plates three times to ensure clonal isolation.

To identify *Curtobacterium* isolates, the 16S rRNA gene was PCR amplified and sequenced. Individual colonies were boiled for 1 min in 50 μL of sterile dH_2_O prior to PCR amplification. Next, 3.0 μL of the boiled bacterial colony was added to the PCR cocktail containing 0.3 μL of Taq polymerase (5 units/μL), 15.0 μL of Premix F (Epicentre, Madison, WI), and 50 μM of each primer in a final volume of 30 μL. We amplified 1500 bp of the 16S rRNA gene using the pA (5′-AGAGTTTGATCCTGGCTCAG-3′) and pH (5′-AAGGAGGTGATCCAGCCGCA-3′) primers (Edwards et al., 1989). Forward and reverse strands were trimmed and merged using Geneious v6.1 (Drummond et al., 2011) under the default parameters. Isolate identity was tentatively assigned using the best-identified match with the blastn alignment (Altschul et al., 1997) within GenBank. In total, 34 Microbacteriaceae isolates were identified, including 17 *Curtobacterium* isolates.

#### Whole genome analysis

This Whole Genome Shotgun project including the genome sequences of 14 *Curtobacterium*, 1 *Frigoribacterium*, and 1 *Plantibacter* isolates deposited at GenBank under BioProject PRJNA342146 with accessions MJGI00000000-MJGX00000000. Paired-end 100 bp × 100 bp whole genome sequencing libraries with a mean gap size of 400 bp were prepared from genomic DNA using the Nextera DNA Library Preparation Kit (Illumina Inc., San Diego, CA, USA). Genomes were sequenced on an Illumina HiSeq 2500 apparatus (Illumina Inc., San Diego, CA, USA) at the Whitehead Institute Genome Technology Core (Cambridge, MA). After quality trimming and removal of short (<30 bp) reads, an initial *de novo* assembly was performed in CLC Genomics Workbench (CLC Bio, Cambridge, MA, USA) using the default parameters.

Genomes (fully assembled and whole genome shotgun assembly) belonging to the Microbacteriaceae were retrieved from the Pathosystems Resource Integration Center (PATRIC) database (Wattam et al., 2013). To annotate these downloaded genomes and our isolate genomes, we first assigned open reading frames (ORFs) sequences as called by Prodigal v2.6 (Hyatt et al., 2010). Genomic ORFs were searched against the Pfam database (Finn et al., 2014) for the presence of protein families using HMMer (Johnson et al., 2010). We identified the GH families as in Berlemont and Martiny (2013) and compiled the number of occurrences of each GH family in each genome. To create a phylogeny of the whole genome sequences, the 16S rRNA region of each genome was predicted using Barrnap^2^. The resulting sequences were used for phylogenetic reconstruction as described above.

## Results

### Geographic distribution of curtobacterium

**Figure 1:**
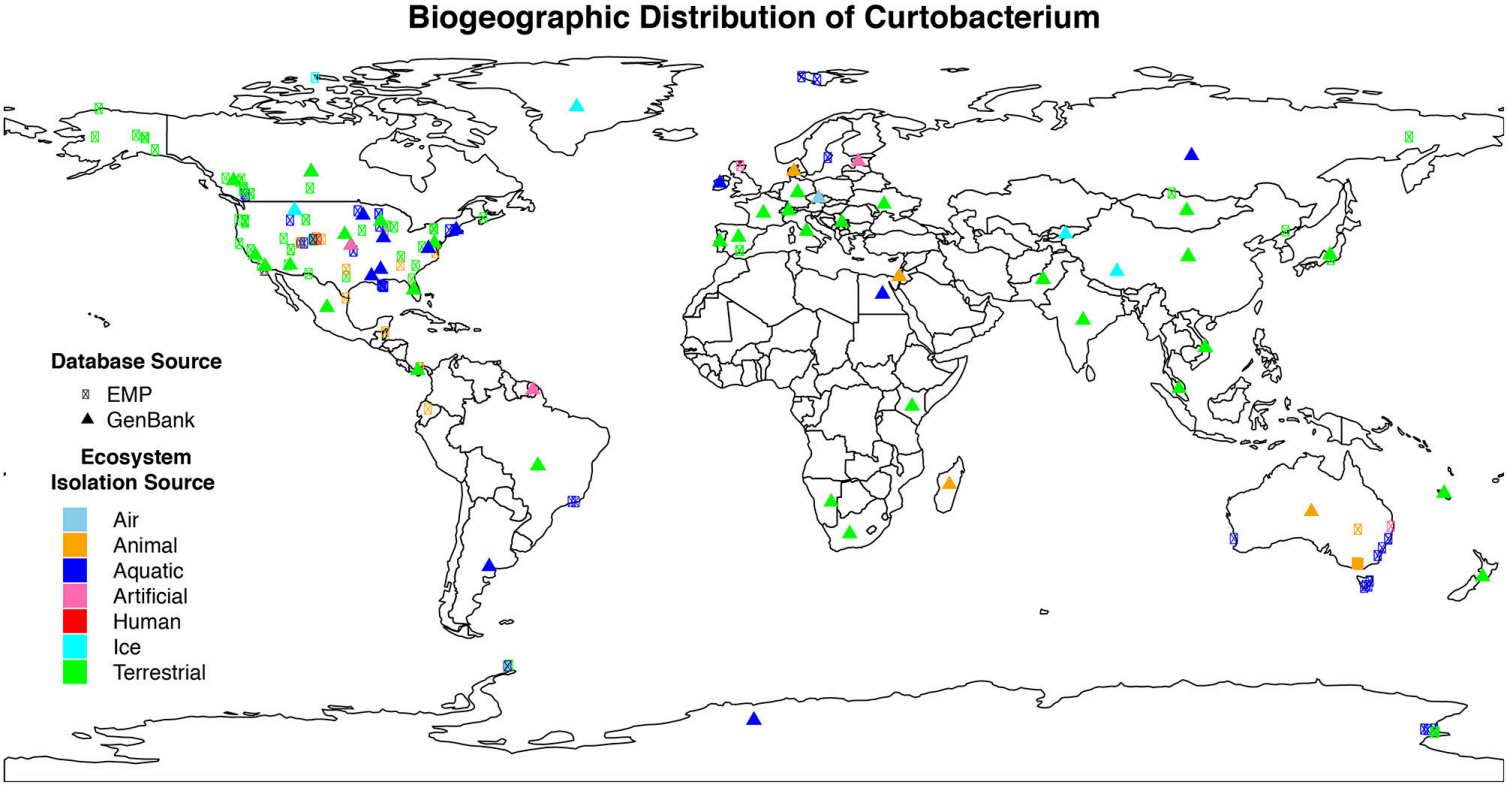
**Geographic distribution of *Curtobacterium* compiled from various isolation sources**. Colors indicate the different ecosystems from which the sequence was isolated. The symbol shape indicates the dataset from which the sequence originated. Sequences obtained from GenBank (triangle symbols) were mostly approximations as detailed GPS coordinates were not available.

We isolated 17 *Curtobacterium* strains from two invasive grassland sites. Although similar in their vegetation, LRGCE and BACE sites are 4130 km apart across the North American continent. Yet, from these sites, *Curtobacterium* strains comprised 10 and 15% of culturable isolates in LRGCE and BACE, respectively. Beyond these two terrestrial sites, data collected from a wide array of studies and isolation sources reveal that *Curtobacterium* is an abundant and globally distributed taxon. In total, we obtained 3360 16S rRNA sequences with corresponding metadata from GenBank and the EMP databases. The genus was found on all continents, ranging from the Arctic to the Antarctic (Figure 1). The majority of sequences were isolated from North America (61.6%), while there was a lack of representation in the Southern hemisphere, most likely due to sampling effort. Australia, South America, Africa, and Antarctica accounted for only 15.3% of all sequences.

*Curtobacterium* has been identified in all designated ecosystems, including animal microbiome, aquatic, artificial, atmosphere, human microbiome, ice, and terrestrial (Supplementary Table 1). The human and animal microbiome comprised 26.9 and 12.9% of all obtained *Curtobacterium* sequences, respectively. *Curtobacterium* sequences from humans were comprised almost exclusively of samples originating from skin, while those from animals were primarily collected from the gut. Most *Curtobacterium* sequences (32.6%) from the EMP dataset were from human microbiome samples, reflecting the emphasis on humans in this dataset. In contrast, only 10.8% of *Curtobacterium* sequences retrieved from GenBank were associated with the human microbiome. After excluding human microbiome samples, over 63% of all sequences originated from terrestrial ecosystems. Specifically, 14% of all sequences were extracted from a plant source and 21% from soil. Sequences from the GenBank database revealed a stronger association with 70.1% of sequences being classified into a terrestrial ecosystem (Supplementary Table 1). Terrestrial samples from the GenBank database included 58.9% from plants and 28.4% from soil.

### Phylogenetic diversity

**Figure 2:**
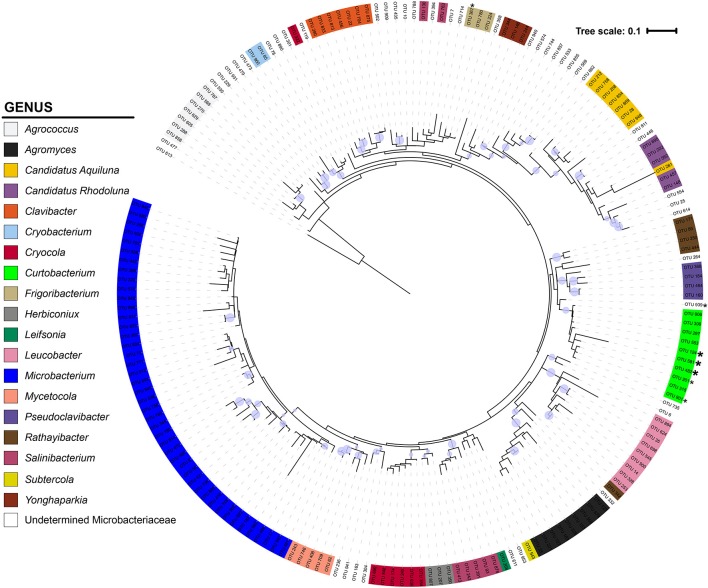
**Phylogeny of Microbacteriaceae constructed from the 16S rRNA gene (maximum likelihood tree with 100 bootstraps and a GTR + GAMMA distribution)**. The tree is color-coded by genus using the taxonomic designation assigned from a combination of SILVA, BLAST, and RDP. The circles represent nodes with at least 70% support and the diameter of the circle represents the support level. ^*^ = 1 litter isolate within the OTU; ^*^ = >4 litter isolates in OTU.

The Microbacteriaceae sequences clustered into 971 OTUs at a 97% similarity level. Considering only OTUs with more than 10 sequence representatives, the remaining 183 OTUs represented 19 genera (Figure 2). The 10 *Curtobacterium* OTUs form a well-supported (bootstrap support of 89%) monophyletic clade. Their closest relatives belong to the *Rathayibacter* and *Pseudoclavibacter* genera. The 17 *Curtobacterium* litter isolates from the Loma Ridge and Boston sites clustered together into five OTUs. Two *Curtobacterium* OTUs contained only one litter isolate despite being in the top 25 of the most abundant OTUs in the SILVA database.

**Figure 3:**
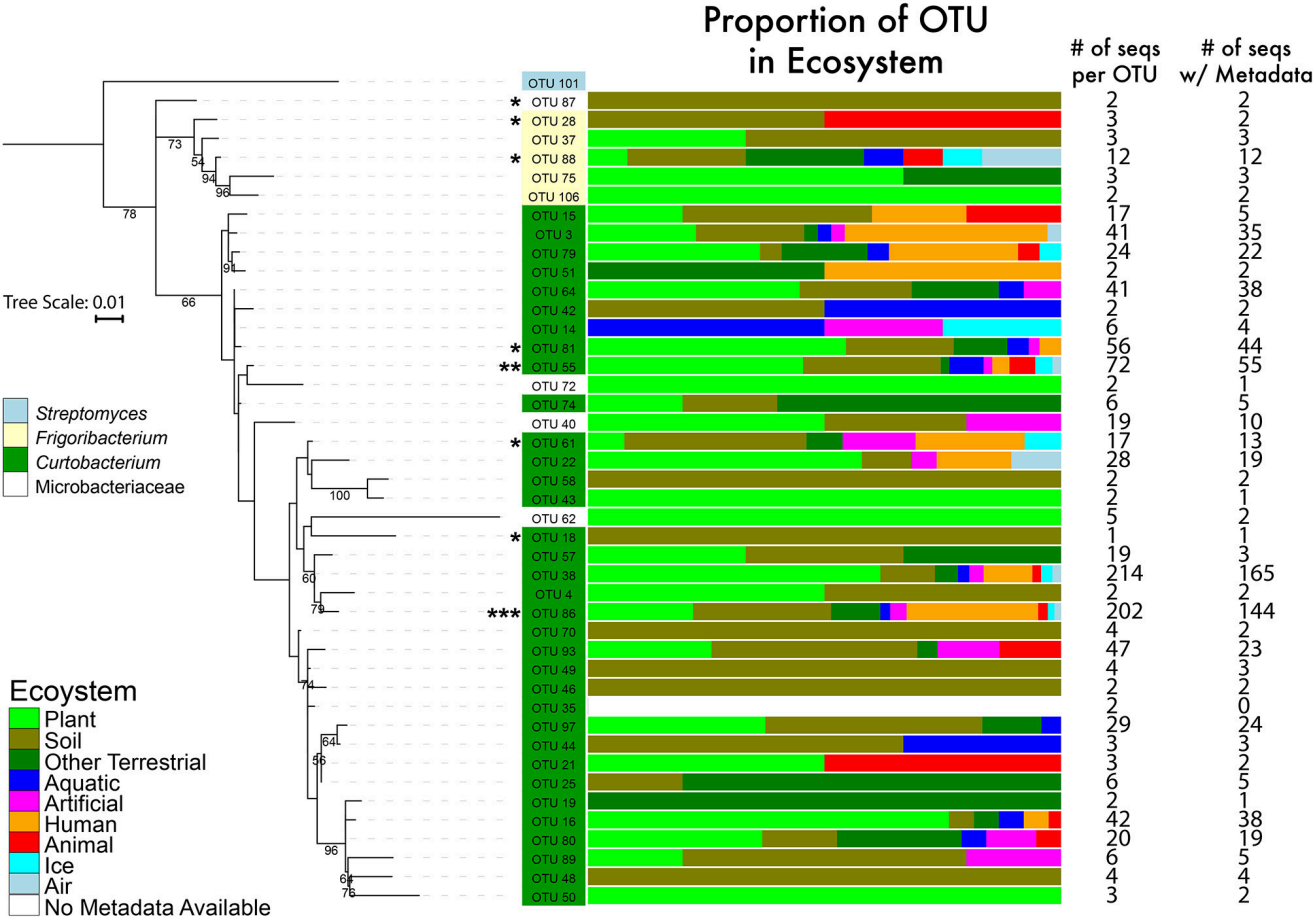
**Phylogenetic tree of the genus *Curtobacterium* with 16S rRNA gene (maximum likelihood tree with 100 bootstraps and a GTR + GAMMA distribution)**. The numbers represent the support level of each node with at least 50% support. Bar graphs are color coded to show the percentages of the OTUs with sequences isolated from various ecosystems. Numbers in the columns represent the number of sequences incorporated into each branch for its respective OTU. ^*^ = 1–4 litter isolates within the OTU; ^**^ = 5 isolates; ^***^ = 10 isolates.

To examine *Curtobacterium* diversity at a finer genetic resolution, we clustered the 1074 total sequences retrieved from GenBank and SILVA with our 22 isolates at a 99% similarity level. This yielded 100 *Curtobacterium* and 7 *Frigoribacterium* OTUs, a sister genus. Excluding singletons, the remaining 52 OTUs represented 1014 *Curtobacterium* sequences with 764 of those sequences containing metadata originating from GenBank entries. Of these sequences, 582 (74%) sequences were isolated from a terrestrial ecosystem. Due to some OTUs containing many sequences without habitat data, distribution of ecosystem preference across phylogeny was not possible. However, there were OTUs detected solely in one ecosystem (e.g., OTU 25 was only found in terrestrial ecosystems), while others OTUs were detected in a variety of ecosystems (e.g., OTU 38 was found in all seven assigned ecosystems). At the level of 99% sequence similarity, most (10 out of 18) of the litter isolates clustered into one abundant OTU (86). This abundant OTU contained over 202 sequences isolated from all seven assigned ecosystems (Figure 3).

### Genomic potential for carbohydrate degradation

Full genomes were used to compare the genomic diversity of glycoside hydrolases within Microbacteriaceae. We included 14 *Curtobacterium*, 1 *Frigoribacterium*, and 1 *Plantibacter* isolates from our leaf litter sites at LRGCE and BACE. The *Curtobacterium* assemblies produced an average genome size of 3.76 Mbp in an average of 78 contigs (mean maximum contig length of 582,567 bp), with an average GC content of 70.47% (Table 1).

**Table 1:** **General characteristics of the litter isolates**.

**Genome ID**	**Taxonomy**	**# of contigs**	**Length (bp)**	**% GC**	**# of ORFs**	**Richness of GH Families**	**# of GHs**
MCBA15_001	*Curtobacterium*	137	3808678	70.12	3940	28	81
MMLR14_002	*Curtobacterium*	40	3634776	71.39	4013	28	79
MCBA15_003	*Curtobacterium*	75	3648432	71.07	3743	29	90
MCBA15_004	*Curtobacterium*	91	3772244	69.38	3633	19	62
MCBA15_005	*Curtobacterium*	26	3601746	72.01	3941	27	77
MMLR14_006	*Curtobacterium*	87	3768639	69.80	3742	29	90
MCBA15_007	*Curtobacterium*	75	4023578	70.40	3888	28	77
MCBA15_008	*Curtobacterium*	41	3649950	71.44	4198	31	103
MCBA15_009	*Curtobacterium*	23	3476500	70.57	3759	29	90
MMLR14_010	*Curtobacterium*	112	3902159	70.56	4034	31	93
MMLR14_011	*Plantibacter*	104	4089281	69.16	4422	25	105
MCBA15_012	*Curtobacterium*	83	3616790	71.04	3638	19	62
MCBA15_013	*Curtobacterium*	86	3948212	69.88	4167	28	83
MMLR14_014	*Curtobacterium*	139	3822836	69.91	4017	27	78
MCBA15_016	*Curtobacterium*	77	3947873	69.07	4103	28	84
MCBA15_019	*Frigoribacterium*	27	3783004	70.04	3633	24	60

*Strains originating from LRGCE are labeled as MMLR, while strains from BACE labeled as MCBA*.

Combining these genomes with the 104 publicly available genomes retrieved from the PATRIC database reveal that strains within Microbacteriaceae contain many diverse GH families. Across the 120 genomes, we identified 7355 potential glycoside hydrolases (GHs) and carbohydrate binding modules (CBMs) representing 63 GH/CBM families (Supplementary Table 2). The most common and ubiquitous families belonged to those targeting starch (GH13, CBM48) and oligosaccharides (GH1, 2, and 3). These GH families were present in most genomes with 92.5% of the genomes containing at least one copy of GH13. GHs that targeted more recalcitrant carbohydrates such as fructan, dextran, mixed polysaccharides, animal polysaccharides, plant polysaccharides, cellulose, chitin, and xylan were also detected in a variety of genomes across Microbacteriaceae, albeit at a lower frequency (Table 2).

**Table 2:** **Breakdown by genus of the distribution of GHs and CBMs by targeted substrate**.

**SUBSTRATE**		***Agreia***	***Agrococcus***	***Agromyces***	***Candidatus Aquiluna***	***Candidatus Rhodoluna***	***Clavibacter***	***Cryobacterium***	***Cryocola***	***Curtobacterium***	***Frigoribacterium***	***Glaciibacter***	***Gulosibacter***	***Herbiconix***	***Humibacter***	***Leifsonia***	***Leucobacter***	***Plantibacter***	***Microbacterium***	***Mycetocola***	***Pseudoclavibacter***	***Rathayibacter***	***Salinibacterium***	***Zimmermannella***	**TOTAL**
**# of Genomes/Genus**		1	2	4	1	1	8	2	1	20	2	1	1	1	1	6	10	1	49	2	1	3	1	1	120
Oligosaccharide	Richness of GHs	4.0	1.0	6.0	−	−	5.0	3.0	6.0	6.0	5.0	5.0	3.0	4.0	5.0	6.0	3.0	5.0	6.0	5.0	−	1.0	4.0	1.0	6.0
	Average # of GHs	4.8	0.2	3.5	−	−	3.5	1.0	5.7	3.3	2.2	5.0	0.7	3.0	5.7	4.3	0.4	7.5	3.8	4.0	−	0.2	2.5	0.2	3.1
	GHs per genome	29.0	1.0	21.3	−	−	20.8	6.0	34.0	20.9	13.0	30.0	4.0	18.0	34.0	26.0	2.1	45.0	22.6	24.0	−	1.0	15.0	1.0	18.6
Starch	Richness of GHs	3.0	3.0	3.0	1.0	2.0	3.0	4.0	3.0	3.0	3.0	3.0	3.0	2.0	3.0	3.0	2.0	3.0	5.0	1.0	−	3.0	2.0	3.0	5.0
	Average # of GHs	3.8	3.0	1.3	0.2	2.0	3.4	3.8	3.6	3.6	3.7	1.8	2.2	1.4	2.6	2.4	0.1	2.6	2.7	0.8	−	2.2	0.8	2.2	2.5
	GHs per genome	19.0	15.0	6.3	1.0	10.0	16.9	19.0	18.0	17.5	18.5	9.0	11.0	7.0	13.0	12.2	0.7	13.0	13.4	4.0	−	11.0	4.0	11.0	12.6
O.A.P.	Richness of GHs	2.0	−	4.0	−	−	1.0	3.0	1.0	2.0	1.0	2.0	−	1.0	1.0	3.0	−	−	4.0	1.0	−	1.0	1.0	−	4.0
	Average # of GHs	1.3	−	0.9	−	−	1.0	1.0	1.3	1.5	0.5	0.8	−	0.5	1.5	1.0	−	−	0.8	0.3	−	0.5	1.0	−	0.8
	GHs per genome	5.0	−	3.5	−	−	4.0	4.0	5.0	5.7	2.0	3.0	−	2.0	6.0	4.0	−	−	3.1	1.0	−	2.0	4.0	−	3.2
O.P.P.	Richness of GHs	3.0	−	7.0	−	−	6.0	4.0	5.0	7.0	7.0	3.0	−	1.0	3.0	6.0	2.0	3.0	9.0	4.0	−	1.0	1.0	2.0	10.0
	Average # of GHs	1.4	−	0.8	−	−	0.5	0.3	1.1	1.0	0.7	0.9	−	0.1	0.6	6.3	0.1	1.4	0.7	0.6	−	0.1	0.2	0.3	0.6
	GHs per genome	14.0	−	8.0	−	−	5.1	3.0	11.0	10.0	6.5	9.0	−	1.0	6.0	6.3	0.5	14.0	6.7	6.0	−	1.0	2.0	3.0	6.1
Mixed Polysacc.	Richness of GHs	4.0	1.0	7.0	−	−	3.0	2.0	3.0	6.0	2.0	6.0	1.0	5.0	3.0	6.0	1.0	4.0	11.0	2.0	1.0	1.0	1.0	1.0	11.0
	Average # of GHs	1.7	0.7	0.8	−	−	0.2	0.5	1.2	0.8	0.7	0.9	0.3	0.9	1.2	0.5	0.3	1.5	1.1	0.3	0.3	0.3	0.3	0.3	0.8
	GHs per genome	19.0	7.5	9.0	−	−	2.4	5.5	13.0	9.4	8.0	10.0	3.0	10.0	13.0	5.5	3.0	16.0	12.0	3.0	3.0	3.0	3.0	3.0	8.6
Cellulose	Richness of GHs	2.0	−	3.0	−	−	2.0	3.0	4.0	5.0	3.0	−	−	−	2.0	5.0	−	4.0	8.0	1.0	−	−	−	−	8.0
	Average # of GHs	0.4	−	0.3	−	−	0.3	0.3	0.8	0.5	0.6	−	−	−	0.5	0.4	−	0.6	0.3	0.3	−	−	−	−	0.3
	GHs per genome	3.0	−	2.0	−	−	2.8	2.0	6.0	4.1	5.0	−	−	−	4.0	3.2	−	5.0	2.1	2.0	−	−	−	−	2.2
Xylan	Richness of GHs	−	−	2.0	−	−	1.0	−	−	2.0	−	−	−	−	−	−	−	1.0	2.0	−	1.0	−	−	−	3.0
	Average # of GHs	−	−	0.3	−	−	1.0	−	−	0.2	−	−	−	−	−	−	−	0.3	0.1	−	0.3	−	−	−	0.2
	GHs per genome	−	−	1.0	−	−	2.9	−	−	0.7	−	−	−	−	−	−	−	1.0	0.3	−	1.0	−	−	−	0.5
Chitin	Richness of GHs	1.0	−	2.0	−	−	1.0	2.0	2.0	2.0	1.0	2.0	−	3.0	1.0	2.0	3.0	−	4.0	−	−	−	1.0	2.0	4.0
	Average # of GHs	0.5	−	1.0	−	−	0.2	1.4	1.5	0.8	0.3	0.8	−	3.5	0.8	0.7	0.4	−	0.3	−	−	−	0.5	0.8	0.5
	GHs per genome	2.0	−	4.0	−	−	0.8	5.5	6.0	3.1	1.0	3.0	−	14.0	3.0	2.8	1.7	−	1.3	−	−	−	2.0	3.0	1.9
Total	Richness of GHs	19.0	5.0	34.0	1.0	2.0	22.0	21.0	24.0	33.0	22.0	21.0	7.0	16.0	18.0	31.0	11.0	20.0	49.0	14.0	2.0	7.0	10.0	9.0	63.0
	Average # of GHs	22.0	6.0	18.0	2.0	4.0	21.1	18.5	28.0	26.9	24.0	24.0	9.0	17.0	21.0	20.7	4.5	21.2	18.0	25.0	3.0	8.0	11.0	10.0	19.4
	GHs per genome	97.0	24.5	62.0	5.0	17.0	22.0	51.0	114	80.5	60.0	74.0	22.0	57.0	92.0	74.3	8.7	69.1	51.0	105	5.0	19.0	34.0	22.0	61.3

*O.A.P., other animal polysaccharides; O.P.P., other plant polysaccharides*.

GH content was highly variable across genera. Some genera appeared not able to process any structural polysaccharides (cellulose, chitin, or xylan) and constrained to the targeting of oligosaccharides and starch (see Table 2). Others, like *Pseudoclavibacter*, lacked any of the identified GH families that process simpler substrates such as starch, and, presumably, are only capable of processing more complex carbohydrates. A few genera had the genomic potential to digest all identified substrates. Specifically, *Curtobacterium* appeared capable of targeting all substrates at a frequency almost double the family average, particularly with regard to structural polysaccharides. Individual strains with the potential to breakdown and digest all three structural polysaccharides appeared to be restricted within the genera *Curtobacterium* (*N* = 11 genomes; including 8 litter isolates), *Clavibacter* (*N* = 6 genomes), and *Microbacterium* (*N* = 6 genomes).

**Figure 4:**
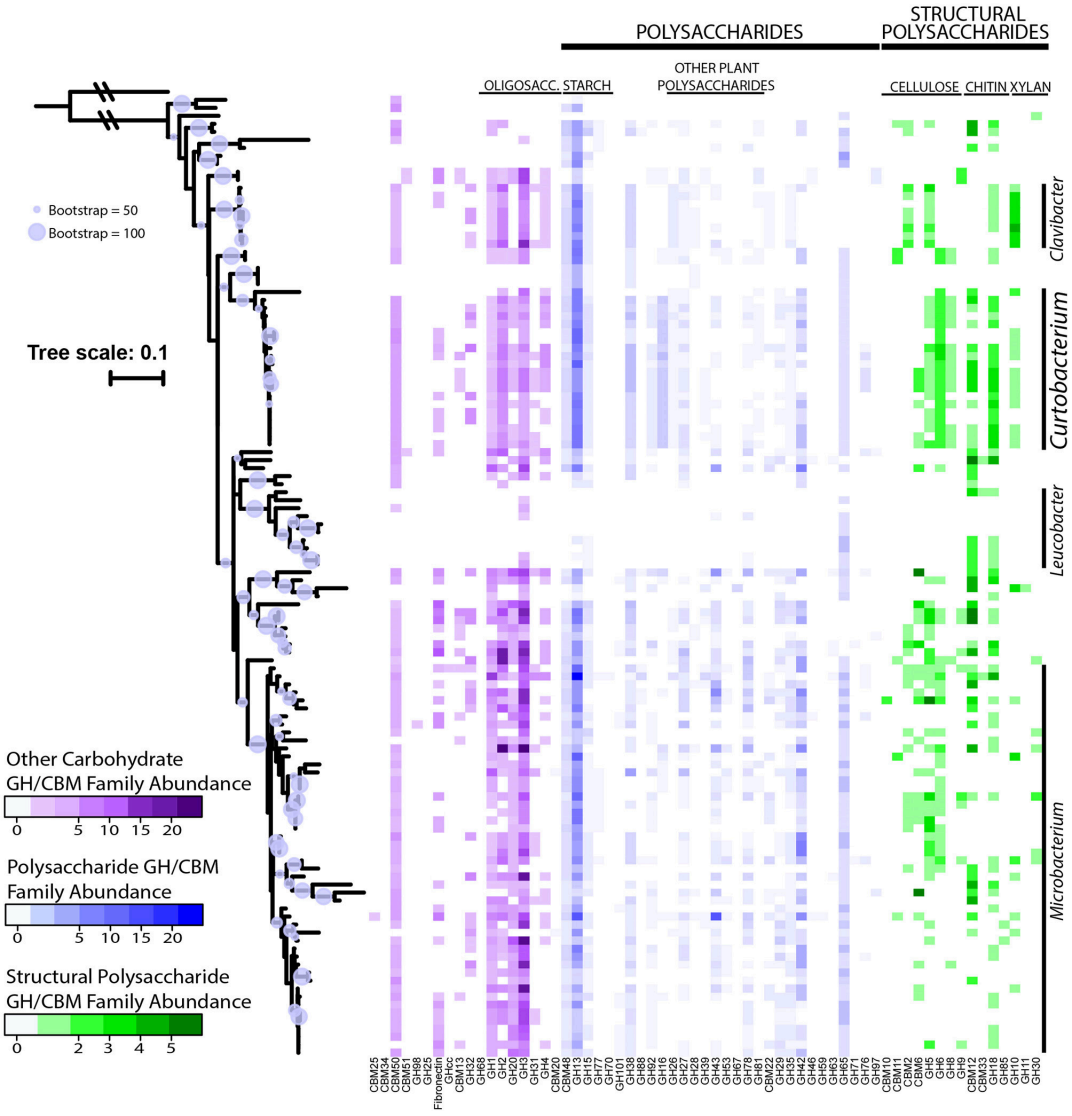
**Abundance of GH and CBM families grouped and colored by substrate category across downloaded Microbacteriaceae genomes from the PATRIC database**. Phylogenetic tree constructed from the 16S rRNA gene sequence (maximum likelihood tree with 100 bootstraps and a GTR + GAMMA distribution). The circles represent nodes with at least 50% support and the diameter of each circle represents the support level. Genera with more than 5 strains are denoted on the right.

The average richness of GH families present in a Microbacteriaceae genome was 19.3 GH/CBM families (Table 2). However, GH/CBM richness varied widely across genomes; a *Leucobacter* genome contained only 1 GH family while one *Microbacterium* species, *Microbacterium sp. SUBG005* (accession number JNNT00000000), had 35 GH families. The litter isolates belonging to *Curtobacterium* had an above average richness of 27.2 GH families with a range of 19–31 GH families. Further, most genomes harbored multiple copies of each protein family. For example, a *Microbacterium* genome had as many as 24 copies of the GH13 family. Due to the multiple GH copies, genomes varied in the total number of GHs present (mean number of GHs = 61.3), ranging from 3 GH proteins in a *Leucobacter* strain to 135 GH proteins in an *Agromyces* strain. On average, the *Curtobacterium* litter isolates encoded 82.1 GH proteins, almost 1.5 times the family average (Figure 4).

We examined the potential for each individual genome to target multiple polysaccharides. Almost all genomes within the family had the potential to process oligosaccharides or starch with the exception of 2 genomes, a *Leucobacter* and *Pseudoclavibacter* strain. Further, a majority of the genomes (103 genomes or 85.8%) within Microbacteriaceae were capable of processing at least one structural polysaccharide. Specifically, the frequency to be able to target cellulose, chitin, and xylan occurred in 64.2, 64.2, and 29.2% of the genomes, respectively.

## Discussion

In this study, we present the first global survey of *Curtobacterium* and show that it is ubiquitous in a variety of ecosystems (Figure 1) although it is most abundant in terrestrial ecosystems, and a majority of sequences are associated with plants and soil. This observation is in accordance with past studies of *Curtobacterium* that attribute its habitat to plants and the related phyllosphere (Komagata et al., 1965; Behrendt et al., 2002). However, *Curtobacterium* is primarily known as a plant pathogen and yet, the highest proportion of *Curtobacterium* strains resided in soil systems, suggesting that this genus may be capable of reproducing in soil.

We also provide a well-supported phylogeny of all known Microbacteriaceae genera. We built upon previous Microbacteriaceae phylogenetic analyses (see Evtushenko and Takeuchi, 2006) to incorporate all available Microbacteriaceae 16S rRNA sequences, providing the most comprehensive phylogenetic analysis of Microbacteriaceae to date (Figure 2). To explore diversity within *Curtobacterium*, we constructed a genus-specific tree to investigate the possibility of clade-specific habitat preference. Due to differences in sequencing platforms and targeted regions of the 16S rRNA gene, there may be habitat specialization at finer clade levels than we are able to differentiate here. In particular, the shorter sequenced reads (e.g., from the EMP dataset) are limited in their phylogenetic resolution and cannot resolve intrageneric patterns. Further, many GenBank sequences lacked metadata altogether or were limited in their details to allow for finer habitat designations (e.g., which part of the plant or the layer of soil from which a strain was isolated). Although we did not detect any clade-specific patterns of habitat preferences, most clades contained a majority of plant and soil isolated sequences (Figure 3), indicating that the genus as a whole may be adapted to plant or soil habitats.

*Curtobacterium* falls within the Actinobacteria phylum, which is known to play a crucial role in the recycling of organic material by decomposition and humus formation (Goodfellow and Williams, 1983). This characterization is supported by a comprehensive analysis into the distribution of GHs across all bacteria, which showed that Actinobacteria has the highest genomic potential for being cellulose degraders (Berlemont and Martiny, 2015). Therefore, we concentrated on these GH proteins, as they are responsible for the breakdown of large carbohydrates that may prove advantageous in decomposition of plant debris. For instance, an increase in diversity and abundance of GHs with the potential for cellulose utilization generally corresponds to better cellulose degradation (Fontes and Gilbert, 2010; Wilson, 2011; Berlemont and Martiny, 2015). Previously, *Curtobacterium* isolates collected from a neutral garden soil were shown to rapidly degrade cellulose fibers (Lednická et al., 2000). Indeed, our results provide a genomic underpin for *Curtobacterium* to be a degrader. The genus has an elevated richness and abundance of GHs relative to other Microbacteriaceae genera. While there is large variation within the family with respect to GH richness and substrate degradation, *Curtobacterium* is one of only three genera with the potential ability to target all identified carbohydrate substrates. Moreover, out of these three genera, *Curtobacterium* has the highest abundance of GHs, suggesting an increased ability to utilize and degrade a wide range of carbohydrates. This variability in carbon usage within *Curtobacterium* suggests that alternative, intrageneric ecological roles have yet to be identified.

We conclude that *Curtobacterium* may be a dominant player in the functional breakdown of dead organic material in leaf litter communities based on its dominance in two grassland litter microbial communities, its high representation in soils, and its genomic potential for being a degrader. This work supports previous studies that show that *Curtobacterium* has the capability to survive on litter (Silva Junior et al., 2012) and thrive as a cellulytic bacterium (Lednická et al., 2000). The conclusion also aligns with culture work that finds that coryneform bacterium, such as *Curtobacterium*, are in high abundance on grasses (Behrendt et al., 2002). Despite the focus in the literature on its role as a crop plant pathogen, future research into the contribution of *Curtobacterium* to the recycling of nutrients in terrestrial ecosystems warrants further attention.

## Author contributions

AC and JM: Developed and designed the study; AC: Preformed data analysis with JM contributing to data interpretation; RB: Assisted in analysis of carbohydrate data; PA and MP: Prepared, assembled, and annotated the genome libraries; AC: Wrote the manuscript with input from all authors.

## Funding

Funding was provided by the US Department of Energy, Office of Science, Office of Biological and Environmental Research (BER), under Award Number DE-PS02-09ER09-25 and by the U.S. National Science Foundation (DEB-1457160) to JM. This work was supported by the U.S. Department of Energy (DE-SC0008743) to MP.

### Conflict of interest statement

The authors declare that the research was conducted in the absence of any commercial or financial relationships that could be construed as a potential conflict of interest.

## Footnotes

^1^https://developers.google.com/public-data/docs/canonical/countries_csv

^2^http://www.vicbioinformatics.com/software.barrnap.shtml
