# Supplementary material for: Evidence for Ecological Flexibility in the Cosmopolitan Genus Curtobacterium
Source: Front Microbiol. 2016 Nov 22;7:1874. doi: 10.3389/fmicb.2016.01874 (PMC5118839; doi:10.3389/fmicb.2016.01874)
Supplement: Supplementary file 1 [file DataSheet1.DOCX]

Supplementary Material

**Evidence for ecological flexibility in the cosmopolitan genus *Curtobacterium***

**Alexander B. Chase^1*^, Philip Arevalo^2^, Martin F. Polz^2^, Renaud Berlemont^3^, and Jennifer B.H. Martiny^1^**

Supplementary Table 1. Metadata associated with collected *Curtobacterium* samples derived from GenBank and the Earth Microbiome Project databases. Samples were manually grouped into ecosystems based on the isolation source of sample.

| **Ecosystem** | **All Databases** | | **GenBank** | | **Earth Microbiome Project** | |
| --- | --- | --- | --- | --- | --- | --- |
|  | **Count** | **% of Samples** | **Count** | **% of Samples** | **Count** | **% of Samples** |
| Animal Microbiome | 433 | 12.9% | 40 | 4.6% | 393 | 15.9% |
| Aquatic | 277 | 8.3% | 56 | 6.4% | 221 | 9.0% |
| Artificial | 142 | 4.2% | 34 | 3.9% | 108 | 4.4% |
| Atmosphere | 15 | 0.4% | 15 | 1.7% | 0 | 0.0% |
| Human Microbiome | 900 | 26.9% | 95 | 10.8% | 805 | 32.6% |
| Ice | 37 | 1.1% | 22 | 2.5% | 15 | 0.6% |
| Terrestrial | 1542 | 46.1% | 615 | 70.1% | 927 | 37.5% |
| **TOTAL** | **3346** |  | **877** |  | **2469** |  |

Supplementary Table 2. List of glycoside hydrolase (GH) families and carbohydrate binding modules (CBMs), associated protein family (Pfam) IDs, and the expected target substrate.

| **GH** | **PfamID** | **Substrate** | **CBM** | **PfamID** | **Substrate** |
| --- | --- | --- | --- | --- | --- |
| GH1 | PF00232 | Oligosaccharides | CBM1 | PF00734 | NA |
| GH2 | PF00703 | Oligosaccharides | CBM2 | PF00553 | cCellulose |
|  | PF02836 | | CBM3 | PF00942 | NA |
|  | PF02837 | | CBM6 | PF03422 | cCellulose |
| GH3 | PF00933 | Oligosaccharides | CBM9_1 | PF06452 | cXylan |
|  | PF01915 | | CBM10 | PF02013 | cCellulose |
| GH4 | PF02056 | Oligosaccharides | CBM11 | PF03425 | cCellulose |
|  | PF11975 | | CBM12 | PF02839 | cChitin |
| GH5 | PF00150 | Cellulose | CBM12 | PF14600 | cChitin |
| GH6 | PF01341 | Cellulose | CBM13 | PF00652 | NA |
| GH7 | PF00840 | NA | CBM14 | PF01607 | NA |
| GH8 | PF01270 | Cellulose | CBM15 | PF03426 | NA |
| GH9 | PF00759 | Cellulose | CBM17/28 | PF03424 | NA |
| GH10 | PF00331 | Xylan | CBM18 | PF00187 | NA |
| GH11 | PF00457 | Xylan | CBM19 | PF03427 | NA |
| GH12 | PF01670 | Cellulose | CBM20 | PF00686 | cStarch / Glycogen |
| GH13 | PF00128 | Starch / Glycogen | CBM21 | PF03370 | NA |
| GH14 | PF01373 | Starch / Glycogen | CBM22 | PF02018 | cMixed Polysaccharides |
| GH15 | PF00723 | Starch / Glycogen | CBM25 | PF03423 | NA |
| GH16 | PF00722 | Other Plant Polysaccharides | CBM27 | PF09212 | NA |
| GH17 | PF00332 | NA | CBM33 | PF03067 | cChitin |
| GH18 | PF00704 | Chitin | CBM34 | PF02903 | NA |
| GH19 | PF00182 | Chitin | CBM35 | PF16990 | cXylan |
| GH20 | PF00728 | Oligosaccharides | CBM40 | PF02973 | NA |
| GH20 | PF02838 | Oligosaccharides | CBM48 | PF02922 | cStarch / Glycogen |
| GH25 | PF01183 | Lysozyme | CBM49 | PF09478 | NA |
| GH26 | PF02156 | Other Plant Polysaccharides | CBM50 | PF01476 | NA |
| GH27 | PF02065 | Other Plant Polysaccharides | CBM51 | PF08305 | NA |
| GH28 | PF00295 | Other Plant Polysaccharides | CBMX | PF06204 | NA |
| GH29 | PF01120 | Mixed Polysaccharides | CBMX2 | PF03442 | cCellulose |
| GH30 | PF02055 | Xylan | Fibronectin | PF00754 | NA |
| GH31 | PF01055 | Oligosaccharides |  |  |  |
| GH32 | PF08244 | Fructan |  |  |  |
|  | PF00251 | |  |  |  |
| GH35 | PF01301 | Mixed Polysaccharides |  |  |  |
| GH38 | PF01074 | Other Animal Polysaccharides |  |  |  |
|  | PF07748 | |  |  |  |
| GH39 | PF01229 | Other Plant Polysaccharides |  |  |  |
| GH42 | PF02449 | Mixed Polysaccharides |  |  |  |
|  | PF08533 | |  |  |  |
|  | PF08532 | |  |  |  |
| GH43 | PF04616 | Other Plant Polysaccharides |  |  |  |
| GH44 | PF12891 | Cellulose |  |  |  |
| GH45 | PF02015 | Cellulose |  |  |  |
| GH46 | PF01374 | Mixed Polysaccharides |  |  |  |
| GH47 | PF01532 | NA |  |  |  |
| GH48 | PF02011 | Cellulose |  |  |  |
| GH49 | PF03718 | Mixed Polysaccharides |  |  |  |
| GH52 | PF03512 | NA |  |  |  |
| GH53 | PF07745 | Other Plant Polysaccharides |  |  |  |
| GH56 | PF01630 | NA |  |  |  |
| GH57 | PF03065 | Starch / Glycogen |  |  |  |
| GH59 | PF02057 | Mixed Polysaccharides |  |  |  |
| GH61 | PF03443 | NA |  |  |  |
| GH62 | PF03664 | NA |  |  |  |
| GH63 | PF03200 | Mixed Polysaccharides |  |  |  |
| GH65 | PF03633 | Mixed Polysaccharides |  |  |  |
|  | PF03632 | |  |  |  |
|  | PF03636 | |  |  |  |
| GH66 | PF13199 | Dextran |  |  |  |
| GH67 | PF07477 | Other Plant Polysaccharides |  |  |  |
|  | PF07488 | |  |  |  |
|  | PF03648 | |  |  |  |
| GH68 | PF02435 | Fructan |  |  |  |
| GH70 | PF02324 | Dextran |  |  |  |
| GH71 | PF03659 | Mixed Polysaccharides |  |  |  |
| GH72 | PF03198 | NA |  |  |  |
| GH75 | PF07335 | Mixed Polysaccharides |  |  |  |
| GH76 | PF03663 | Mixed Polysaccharides |  |  |  |
| GH77 | PF02446 | Starch / Glycogen |  |  |  |
| GH78 | PF05592 | Other Plant Polysaccharides |  |  |  |
| GH79 | PF03662 | NA |  |  |  |
| GH80 | PF13647 | NA |  |  |  |
| GH81 | PF03639 | Other Plant Polysaccharides |  |  |  |
| GH85 | PF03644 | Chitin |  |  |  |
| GH88 | PF07470 | Other Animal Polysaccharides |  |  |  |
| GH92 | PF07971 | Other Animal Polysaccharides |  |  |  |
| GH97 | PF10566 | Mixed Polysaccharides |  |  |  |
| GH98 | PF08307 | NA |  |  |  |
|  | PF08306 | |  |  |  |
| GH100 | PF12899 | Mixed Polysaccharides |  |  |  |
| GH101 | PF12905 | Other Animal Polysaccharides |  |  |  |
| GH108 | PF05838 | Mixed Polysaccharides |  |  |  |
| GHcc | PF11790 | NA |  |  |  |
